# The Plea of the Patient

**Published:** 1888-09-15

**Authors:** 


### THE PLEA OF THE PATIENT.

When an ordinary man sits down to dinner he does not ask whether the bullock was fed in Aberdeenshire or in Kent, nor is he curious to know if the cook was trained in Whitechapel or in South Kensington: the two points about which he is chiefly if not entirely interested are in the quality of the sirloin and the excellence of the cooking. Will medical men, deans of medical schools, and medical students permit a word of direct address? When a man is ill in bed, with some mortal disease, say typhoid fever, does he care one atom whether his medical attendant obtained first-class honours or only second in some school competition, or whether he is M.D. London or M.D. Borneo? Not one straw! What he does care about, and what his friends care about, is whether his experience and knowledge are such as to enable him to manage the case in hand with the wisest possible judgment, and whether he is so kind and so honourable that they may rely upon him to treat it exactly as he himself would desire to be treated if he were compelled to change places with his patient. Of course no one with the least degree of sense wishes to disparage intellectual and scientific training. On the contrary, it is to be encouraged in every legitimate way. But what the patient feels is that though such training is desirable in a high degree, the practical knowledge, the ripe judgment, and the genuine kindness of the ideal medical man are essential and indispensable. In all things there must be order and measure. To put that first which should be second, or even fourth; and to put that fourth which should be first, does not end with merely proving stupidity on the part of the master of the ceremonies; it results in practical injury, and in the case of medical men may prove the death of many patients. Much, therefore, as a patient may admire science in the abstract, and proud as he may be of its progress in his own times, he still desires in the practising doctor, as the chief things, knowledge of bedside work, practical skill, and kindness. This should be the first and paramount object which medical schools have in view in their manipulation of medical students. It is proper to point out that some means ought to be employed to discover beforehand whether the candidate for registration has those natural aptitudes of character which will fit him to play his part at the bedside with comfort and advantage to his patient and with honour to himself. The duties of medical men place them often in most delicate and trying circumstances with both sexes. Some doctors—a good many, in fact—are more fitted for the plough and the scythe than for the bedside, whilst not a few would find congenial occupation as tavern waiters or professional pugilists. There is practically no barrier to the medical profession, except those of failure to pay fees and to pass examinations. If the sentinels who guard the portals were equal to their duties, there would be a barrier of unfitness on the ground of temperament and moral character. The heads and controllers of medical schools entirely fail of their duty, and indeed show themselves unfit for their positions, if they do not steadily keep before the medical student, from his first year to his last, this ideal of perfect bedside practice. They have the power to mould the average youth almost entirely as they please. On them more than on any one else depends the kind of practitioner to whom the family confidence must be entrusted, and in whose competent or incompetent hands must be placed the family life and limb. A word to the student may properly end this plea. He should feel even more keenly than his teachers that his life in hospital is a crucial time for him. He is laying the foundation of his future fortunes. He ought to wish, and the best students do wish so to use their opportunities in the medical school as to make themselves in the highest degree worthy of trust and confidence as practitioners in their future life. To a student who knows what the noblest motives are, little needs to be said. But those who enter upon medicine with the commonplace motive of earning a living in a gentlemanly kind of way should remember, even as a matter of business, that the very first of all considerations for them should be that of fitting themselves for thoroughly competent practice at the bedside. If they wish to become men of science, and to follow science purely, by all means let them give themselves up to it entirely. But if their ultimate destination is medical practice, they should study science and everything else as means to the principal end. From the patient's point of view a man must be a doctor first of all, and a kind and competent doctor, whatever he be in his leisure and at home.

